# Introduction to network meta-analysis: understanding what it is, how it is done, and how it can be used for decision-making

**DOI:** 10.1093/aje/kwae260

**Published:** 2024-08-06

**Authors:** Romina Brignardello-Petersen, Gordon H Guyatt

**Affiliations:** Department of Health Research Methods, Evidence, and Impact; McMaster University, Hamilton, Ontario, Canada; Department of Health Research Methods, Evidence, and Impact; McMaster University, Hamilton, Ontario, Canada

**Keywords:** network meta-analysis, multiple comparisons, systematic reviews

## Abstract

Network meta-analysis (NMA), a statistical technique that allows systematic reviewers to simultaneously compare more than 2 alternatives, makes use of indirect evidence from studies comparing interventions of interest to a common comparator. The capacity for multiple simultaneous comparisons makes NMA appealing for evidence-based decision-makers. This article, aimed at users of systematic reviews (SRs) with NMAs and at those who are considering conducting SRs with NMAs, provides an introductory level overview of this topic. We describe the main considerations that those conducting systematic reviews with NMA should bear in mind, including decisions regarding grouping interventions into analysis nodes, and testing the assumptions that assure the validity of NMA. We explain and illustrate how both systematic reviewers and users should draw conclusions from NMA that are appropriate and useful for decision-making. Finally, we provide a list of tools that facilitate the conduct and interpretation of NMAs.

## Introduction

For most healthcare questions addressing the effects of preventive or therapeutic interventions, there are more than 2 alternatives to consider. For instance, those wondering what is the most effective nutraceutical for weight loss in adults who are overweight or obese may consider green coffee, green tea, flaxseed, capsaicin, spirulina, and up to 13 others.^1^ Similarly, if the goal is identifying the most effective prophylactic interventions for modulating the intestinal microbiome in premature infants and reducing mortality, options include probiotics, prebiotics, lactoferrin, and their combinations.^2^

In a previous article,^3^ we described the importance of using systematic reviews to collect, synthesize, appraise, and summarize the relevant evidence for healthcare questions. A limitation of traditional systematic reviews of interventions, however, is that they focus on the comparison of a pair of interventions rather than the simultaneous comparison of several options. Traditional systematic reviews are thus restricted to evaluating the relative merits of interventions compared in head-to-head trials, often leaving out key comparisons beyond prior existing methods.

Systematic reviews (SRs) with network meta-analysis (NMA) overcome these limitations. This article describes what an NMA is, how it is done, and how readers should interpret and use NMA results to inform decision-making. Existing articles on this topic focus on theoretical aspects,^4^^,^^5^ do not include the latest approaches to use results from NMAs,^6^ or have been written for a very specific audience. To illustrate the concepts, we use examples addressing the effects of clinical interventions for managing public health concerns,^2^^,^^7^ but all the concepts also apply to NMAs addressing questions about the effects public health interventions,^8^^-^^10^ questions about risk and prognostic factors,^11^^,^^12^ and questions about diagnosis.^13^^,^^14^ Users of SRs with NMAs and those who are considering conducting SRs with NMAs are likely to find this introductory level overview of this topic of use.

## What is NMA?

Network meta-analysis is a statistical technique that informs decision-makers’, clinicians’, and patients’ choices when they face several options to deal with a healthcare problem. Network meta-analysis is an extension of traditional meta-analysis that incorporates 3 or more interventions. Network meta-analysis provides an approach to combining the results of studies that are connected by common comparators. The output includes estimates of the relative effects of all pairs of comparisons in the network (ie, every alternative against every other alternative).^15^

**Figure 1:**
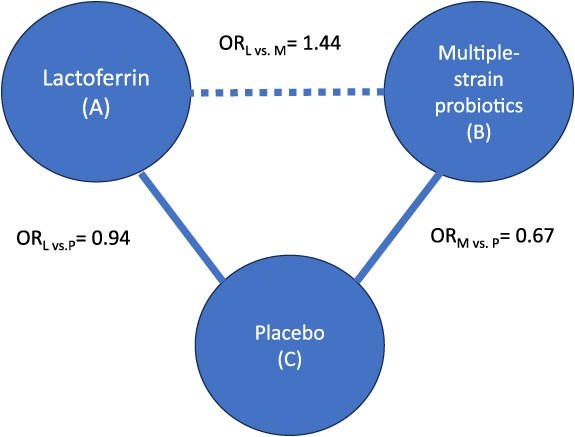
Example of a subset of interventions included in a network meta-analysis (NMA), focusing on the comparison between lactoferrin (A) and multiple-strain probiotics (B) through the common comparator placebo (C), where the outcome of interest is mortality. The solid line represents the availability of studies comparing interventions, and the dashed line represents how NMA allows obtaining an estimate of effect comparing A vs B, through the common comparator C. The odds ratio (OR) comparing A vs C is 0.94 (ie, A reduces mortality), and the OR comparing B vs C is 0.67 (ie, B reduces mortality, and by a larger magnitude than A does). Based on this, the NMA^2^ (which includes more interventions) estimates that the OR comparing A vs B is 1.44 (ie, the risk of mortality is higher with A than B). This figure represents the simplest NMA, with 3 interventions, and its output includes an estimate of the effect of A vs B, A vs C, and B vs C.

To address the relative effects of candidate interventions, NMA uses both direct and indirect evidence. Direct evidence comes from studies that compare the options head-to-head. Indirect evidence originates from sets of studies with a common comparator, which is used to make indirect comparisons. Thus, NMA allows making inferences regarding each possible pairing of available treatments, even when they have not been directly compared in studies. For instance, if we are interested in treatment A vs treatment B, we can make inferences about their relative effect if studies compare A vs C and B vs C. The output of an NMA includes a measure of effect (eg, an odds ratio) summarizing the relative effect of each intervention against every other. Figure 1 illustrates this concept in a subset of an NMA^2^ that includes 3 interventions, where lactoferrin (A) and multiple-strain probiotics (B) have not been compared against each other in studies, but because they have both been compared against placebo (C), an NMA allows learning about how they compare with one another with regards to mortality through an indirect comparison.

**Figure 2:**
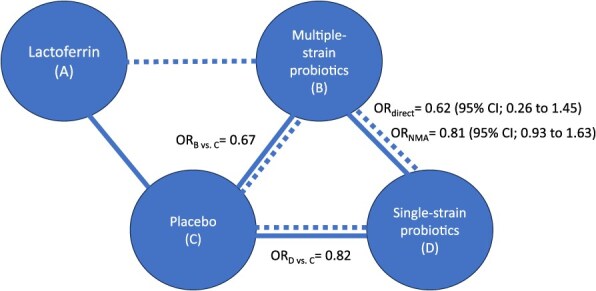
Example of a subset of interventions included in a network meta-analysis, where the outcome of interest is mortality. This figure expands on Figure 1 to include an additional intervention (D) and focuses on the comparison between multiple-strain probiotics (B) and single-strain probiotics (D) for which there is both direct and indirect evidence. The solid line represents the availability of studies comparing B vs D (direct evidence), and the dashed line represents the availability of indirect evidence through the common comparator C. The odds ratio (OR) from the direct evidence is 0.62 (B reduces mortality when compared to D. The indirect comparison confirms that B is more effective than D in reducing mortality (the OR comparing each of them against C shows that B has a larger magnitude of effect). When combining both, the direct and indirect evidence through an NMA, the OR shows that B reduces mortality when compared to D but by a lesser amount than shown by considering only the direct evidence. The addition of indirect evidence through the NMA also reduced the statistical uncertainty represented by the width of the 95% confidence interval.

Network meta-analysis also combines direct and indirect evidence when both are available. That is, if there are studies comparing B vs D (ie, direct evidence), but there is also information through a common comparator such as C (ie, indirect evidence), NMA considers both sources in calculating the effect of B vs D (Figure 2).^2^

### Conducting systematic reviews with NMA

The main steps for conducting a systematic review with NMA are the same as those for conducting a systematic review with a traditional meta-analysis.^3^ Due to the broader scope of the questions NMAs address, however, reviewers should have in mind the following particular considerations:

#### Formulation of the question and eligibility criteria

At this stage, systematic reviewers ensure that they consider all relevant interventions to include in an NMA. This means not only being comprehensive regarding the alternatives that the NMA will compare but also considering if there is any additional common comparator that, even though not of primary interest (eg, an intervention that used to be the standard of care but is no longer commonly used or studied), would provide important indirect evidence. For instance, even if systematic reviewers are only interested in comparing a set of active drugs with one another, if most of these drugs have been compared against placebo or no treatment in studies, reviewers should include studies where placebo or no treatment is the comparator. Otherwise, they risk failing to benefit from indirect evidence that may change their conclusions.

#### Search and selection of studies, and data abstraction and assessment of risk of bias of the included studies

The conduct of the fundamental steps of a systematic review are very similar for traditional meta-analysis and NMA. The only important difference is that when systematic reviewers undertake NMAs, the number of references they screen through and the number of included studies from which they need to abstract data and for which they need to assess risk of bias is often much larger. This larger amount of work results in more time and resources needed to complete a systematic review with an NMA.

#### Data synthesis

This is the stage in which there are the greatest differences between a systematic review with a traditional meta-analysis and one with an NMA. These include:

##### Deciding on treatment nodes

Before they do the analysis, systematic reviewers must decide which treatments they will consider the same vs different. In NMA technical language, they must choose which interventions they will group into a same “node.” Depending on the specific context, reviewers may group interventions according to classes (eg, all classes of antibiotics in the same node) or decide to separate them in different nodes that include any dose of the same drug (eg, all doses of amoxicillin in the same node). For example, a systematic review addressing drug treatments for COVID-19 grouped treatments into nodes based on the specific molecule but not on dose or duration of administration.^7^ Some of the nodes in this NMA included systemic corticosteroids (any), lopinavir-ritonavir (any dose and mode of administration), ivermectin (any dose and mode of administration), and interleukin-6 receptor antagonists (any dose and mode of administration). The choice of lumping treatments into a node or splitting them into different nodes depends on whether reviewers expect the specific drugs or doses to have different effects.

##### Assessing the transitivity assumption

Transitivity is the basis for using indirect comparisons to learn about estimates of effects, and one of the core assumptions of NMA. Transitivity means that the pieces of evidence (direct comparisons) that contribute to an indirect comparison are similar enough that reviewers do not have serious concerns they will obtain a biased indirect estimate.^5^ For example, in Figure 1, the set of trials comparing lactoferrin vs placebo is similar enough to the set of trials comparing multiple-strain probiotics vs placebo with regards to the characteristics of the premature infants included, and the presence of co-interventions, and the overall methods used in the trials. Therefore, the indirect comparison between lactoferrin and multiple-strain probiotics is valid. If there is an important effect modifier in the NMA (eg, infants in the set of trials comparing lactoferrin vs placebo had more risk factors for mortality than those in the set of trials comparing multiple-strain probiotics vs placebo), reviewers should undertake separate analyses according to the levels of the effect modifier (eg, an NMA for infants with lower gestational age and another for infants with higher gestational age).

##### Conducting the statistical analysis

Network meta-analysis is more complex to conduct than traditional meta-analysis. Statisticians have developed methods for NMA conduct in both Bayesian and frequentist frameworks.^16^ Due to the complexity, however, conduct or supervision by a biostatistician or methodologist with ample expertise in NMA is required to ensure sound data analysis.

##### Addressing the coherence assumption

Coherence (also known as consistency), the statistical agreement between direct and indirect evidence, is the second core assumption of NMA.^5^ Incoherence is a measurable manifestation of the lack of transitivity, the other core assumption of NMA. Systematic reviewers should use tests for assessing both global (at the network level) or local (and the level of a loop for which there is direct and indirect evidence) incoherence.^17^^,^^18^ The presence of incoherence may result in reviewers revising their analysis or accounting for this incoherence when assessing the certainty of evidence.^19^

#### Assessing the certainty of evidence and drawing conclusions

Although the process for assessing the certainty of evidence of NMA follows the same principles as for a traditional meta-analysis (ie, it is done for each comparison and outcome separately, it considers the same key domains), the nature of NMAs adds complexity. As described in another article of this series,^20^ in traditional head-to-head comparisons, the Grading of Recommendations Assessment, Development, and Evaluation (GRADE) approach provides a clearly articulated framework to address considerations of risk of bias, inconsistency, imprecision, indirectness, and publication bias to determine how confident systematic reviewers are in the estimate of effect of an intervention.^21^^-^^23^ In addition to the standard certainty rating of each direct estimate informing a network, the GRADE approach addresses issues specific to NMA.^24^^,^^25^ These include the assessment of the certainty of the indirect evidence, including evaluation of transitivity^26^; how much information the direct and indirect evidence contribute to each network estimate; and how coherent (consistent) they are with one another.^19^ Just as in a systematic review with a traditional meta-analysis, these assessments of certainty of evidence are fundamental for drawing conclusions from the systematic review. We provide a detailed description of the principles in the next section.

Despite NMA’s growing popularity (the number of potentially relevant articles retrieved with a specific search in Pubmed Medline increased from under 100 in 2006 to over 500 in 2013,^27^ over 10 000 in February 2020, and 22 167 in March 2024) and the proposal of methods to incorporate the use of different sources of evidence such as clinical trials, observational studies, and real world evidence,^28^^,^^29^ NMA methods are currently mostly applied to address questions regarding the effects of interventions through the synthesis of randomized clinical trials. Because of this, methodological guidance to conduct and interpret NMAs that simultaneously includes different sources of evidence or addresses of other types of questions remains limited to statistical methods.

### Interpreting and using NMA results to inform decision-making

Systematic reviews with NMAs produce a large amount of information. While the smallest network with 3 interventions results in 3 pairwise comparisons with estimates of effect, a larger network with 10 interventions will result in 45 estimates and 15 interventions will result in 105. As the number of interventions in an NMA increases, more challenges in the interpretation of its results arise. An appealing but problematic and potentially misleading feature of NMA is that it allows ranking treatments. That is, it estimates the likelihood of each treatment being the most beneficial (or harmful) for each outcome. It is important to consider, however, that rankings do not convey any information about the magnitude of the effect when comparing interventions ranked adjacently nor about how trustworthy the evidence is,^30^ and that chance could explain differences between ranks.^31^ Therefore, appropriate interpretation of results from NMA requires considering more than the rankings.

Thus, application of results from NMA to clinical practice requires understanding the certainty of the evidence^24^: For some paired comparisons and outcomes the certainty of the evidence may be high, for others very low. Simply looking at the relative effect estimates and the rankings derived only from this statistical information, without considering the certainty of the evidence may result in misleading inferences. For instance, when researchers applied the GRADE approach to an NMA of antidepressants,^32^ they discovered highly variable levels of evidence across the network and therefore concluded that strong inferences about which interventions were more effective and safe were unwarranted: the conclusions should be more conservative.

Therefore, when interpreting results from NMAs and drawing conclusions, systematic reviewers and users must consider both the magnitudes of effect and the certainty of the evidence. As described previously, arriving at accurate interpretations may involve considering a large amount of information. The GRADE working group has developed a solution to this challenge: systematic reviews should categorize the interventions in an NMA in groups, from the most to the least effective (or harmful).^33^^,^^34^ This categorization is based on the principle that, when considering all the available information, rarely will systematic reviewers find that a single intervention is superior to all others. This classification is a more accurate representation of the body of evidence and makes it easier to communicate and understand the results from large networks.^7^^,^^35^^,^^36^

For instance, when summarizing the evidence for the outcome mortality in one of the iterations of the living systematic review and NMA addressing drug treatments for COVID-19,^7^ reviewers included 57 nodes in the NMA. This resulted in 1596 pairwise comparisons for which there was an estimate of effect and certainty of the evidence. Using the principles from the GRADE approach, authors classified the 57 interventions into 5 groups (Table 1) and summarized this extremely large amount of information into a single paragraph and 1 column in a table.^37^

**Table 1:** Classification of the drug treatments for treating people with COVID-19 based on their effects on mortality.^a^

**Category**	**Interventions**
Among the most beneficial, high or moderate certainty	Corticosteroids (systemic) Interleukin-6 receptor antagonists + corticosteroids Jasus Kinases Inhibitors
Not convincingly different than standard care, high or moderate certainty	Hydroxychloroquine Interleukin-6 receptor antagonists
Among the most beneficial, low certainty	Tyrosine kinase inhibitors
Not convincingly different than standard care, low certainty	(Acetyl) cysteine Aspirin Azithromycin Azithromycin + hydroxychloroquine Azithromycin + hydroxychloroquine + oseltamivir Colchicine Fluvoxamine Full-dose anticoagulant Granulocyte-macrophage colony-stimulating factor inhibitor Interleukin-1 inhibitors Interferon beta Intermediate-dose anticoagulant Ivermectin Lopinavir- ritonavir Nitazoxanide Probiotics Remdesivir Sodium-glucose co-transporter 2 inhibitors Synthetic vasoactive intestinal peptide
Very low certainty evidence	Antihypertensive drugs Antihepacvi ral Aspirin + statins Azithromycin + NSAID Azithromycin + NSAID + corticosteroid Cefepime Ceftazidime Colchicine + emtricitabine + tenofovir + statins Colchicine + statins Doxycycline Doxycycline + ivermectin Electrolyzed saline Emtricitabine + tenofovir Favipiravir Favipiravir + hydroxychloroquine Interferon alpha Interferon beta + lopinavir- ritonavir Intranasal corticosteroids Melatonin Methylene blue Molnupiravir Omega 3 Nirmatrelvir/ritonavir Proxalutamide Recombinant human granulocyte colony-stimulating factor Serine protease inhibitors Statins Umifenovir Vitamin C Vitamin D

^a^ Adapted from Siemieniuk et al.^7^ In addition to the classification, in their main results table, the systematic review presents the absolute estimates of effect for each of the interventions when compared with standard care.

### Tools to facilitate conducting NMAs

Systematic reviewers aiming to conduct NMAs addressing questions about the effects of interventions and users interpreting such NMAs can benefit from using several available tools.

#### Tools that provide a description of the methods for conducting systematic reviews with NMAs

The Cochrane Handbook, the main resource that provides details regarding the methodology of all the steps of a SR,^38^ includes a chapter dedicated to NMA.^39^ This chapter describes theoretical considerations and assumptions behind NMA and the particular aspects to which systematic reviewers conducting an NMA must attend.

#### Tools that guide the reporting of systematic reviews with NMA

The Preferred Reporting Items for Systematic Reviews and Meta-Analysis (PRISMA) checklist, which describes the items that systematic reviewers should describe in a publication,^40^ has an extension that focuses on the additional items to make the publication of a systematic review with an NMA transparent and reproducible.^41^ This checklist provides details about the items to consider, but it is not aimed at facilitating the choice of appropriate methods.

#### Tools that aid in the conduct of specific steps of systematic reviews with NMAs

Because NMA is part of a systematic review, the same tools that aid in conducting study searches and selection and abstracting data in a systematic review with a traditional meta-analysis are helpful to those doing NMA. The “Systematic Review Toolbox” (www.systematicreviewtools.com) allows searching for such resources. Reviewers can conduct the statistical analysis in several software, some more user-friendly than others. The Cochrane “Comparing Multiple Interventions” Methods Group provides a list and description of the main software available to conduct NMA.^42^

To aid in the assessment of the certainty of evidence, GRADE NMA experts have published an article in which systematic reviewers can find a detailed description (including a summary in an infographic) of practical considerations and a downloadable spreadsheet that uses automation to facilitate assessments of certainty of evidence using the GRADE approach.^43^ With this same aim, another group of researchers created an online tool that helps systematic reviewers assess their “Confidence in Network Meta-Analysis” through a process that is built using the principles of the GRADE approach.^44^ Although these tools incorporate automation of several steps of the assessment of the certainty of evidence, systematic reviewers should always review the outputs and adjust their final judgments if needed.

#### Tools to assess existing NMAs

Other than several articles written to guide specific medical audiences in using systematic reviews with NMAs,^45^^-^^49^ there is currently no specific tool that can help users in assessing the methodological quality of systematic reviews with NMAs. Researchers are working on developing a tool with this purpose.^50^

## Conclusion

Due to their ability to directly inform decisions that incorporate more than 2 options, systematic reviews with NMA have gained popularity and become more and more common across the scientific healthcare literature. The potential benefits of NMA come at the cost of the added complexity and effort including decisions regarding analysis nodes, implementing the analysis, addressing the NMA core assumptions, and drawing conclusions. It is likely, nevertheless, that NMA will continue to become a much more common method of statistical analysis in systematic reviews. Therefore, to ensure its potential is met, those practicing evidence-based decision-making should understand the underlying methods underlying NMA.
